# Stratified discharge timing within mucositis cases and its association with systemic complications in hematopoietic stem cell transplant recipients: insights from a national inpatient study

**DOI:** 10.1007/s00784-026-07037-w

**Published:** 2026-07-21

**Authors:** Sana Raheem, Kapil Meleveedu, Stephanie J. M. van Leeuwen, Lucky L. A. van Gennip, Lauryn Rudin, Joel B. Epstein, Roberto Pili, Nicole Blijlevens, Poolakkad S. Satheeshkumar

**Affiliations:** 1 Jacobs School of Medicine and Biomedical Sciences, Buffalo, NY 14203 USA; 2 Carole and Ray Neag Comprehensive Cancer Center, University of Connecticut, Farmington, CT 06030 USA; 3 Department of Dentistry, Radboud University Medical Center, Nijmegen, The Netherlands; 4 City of Hope, Duarte, CA USA; 5 Cedars-Sinai Medical System, Los Angeles, CA USA; 6 Department of Medicine, Division of Hematology and Oncology, University at Buffalo, Buffalo, NY 14203 USA; 7 Department of Hematology, Radboud University Medical Center, Nijmegen, The Netherlands

**Keywords:** Oral ulcerative mucositis, Hematopoietic stem cell transplantation, Length of stay, Septicemia, Healthcare-associated complications, Discharge timing

## Abstract

**Background:**

Oral ulcerative mucositis (OUM) is a frequent and severe complication of hematopoietic cell transplantation (HCT). Whether prolonged hospital discharge timing, used as a proxy for extended length of stay (LOS), identifies greater systemic morbidity among patients who develop OUM remains unclear. This retrospective cohort study aimed to evaluate, among HCT recipients with documented OUM, whether prolonged discharge timing is independently associated with increased systemic complications.

**Methods:**

This retrospective cohort study utilized the National Inpatient Sample to identify adult patients with leukemia or multiple myeloma undergoing autologous or allogeneic HCT complicated by OUM. Patients were stratified by discharge timing: autologous HCT, 10–19 (control) vs. 20–29 days; allogeneic HCT, 15–24 (control) vs. 25–34 days. Further, no control group of patients undergoing HCT without OUM was examined. Baseline characteristics and systemic outcomes—including healthcare-associated complications incorporating septicemias, isolated septicemia, coagulopathies, hypertension, and congestive heart failure—were compared. Survey-weighted multivariable logistic regression estimated adjusted odds ratios (aOR) for outcomes in prolonged versus shorter discharge strata, adjusting for demographics and comorbidity burden.

**Results:**

Among autologous HCT recipients with OUM (weighted *n* = 1290), 21.7% experienced prolonged discharge. This stratum had significantly higher adjusted rates of healthcare-associated complications, including septicemias (aOR 3.09, 95% CI: 1.45–6.59), isolated septicemia (aOR 4.68, 95% CI: 1.83–11.96), and congestive heart failure (aOR 1.68, 95% CI: 2.44–11.58). Among allogeneic HCT recipients (weighted *n* = 885), 54.2% had prolonged discharge, associated with increased healthcare-associated complications including septicemias (aOR 3.3, 95% CI: 1.19–9.12), coagulopathies (aOR 3.47 with 95% CI: 1.33–9.03), and hypertension (aOR 2.00 with 95%CI: 1.05–3.82). No adverse outcomes occurred in discharges within 0–10 days.

**Conclusions:**

Prolonged discharge timing in HCT patients with OUM is independently associated with substantial systemic morbidity, including septicemia-related complications. Discharge-based stratification highlights high-risk periods amenable to targeted supportive interventions to mitigate complications and facilitate earlier safe discharge.

**Supplementary Information:**

The online version contains supplementary material available at 10.1007/s00784-026-07037-w.

## Introduction

Hematopoietic stem cell transplantation (HCT) constitutes a potentially curative modality for patients with hematologic malignancies, especially leukemias [1]. Nevertheless, the requisite high-dose conditioning regimens frequently precipitate profound myelosuppression and injury to rapidly proliferating tissues in the oral and gastrointestinal mucosa [2, 3]. Oral ulcerative mucositis (OUM) emerges as one of the most prevalent and debilitating complications, manifesting as inflammation, erythema, and painful ulceration of the oral mucosa [4, 5]. Reported incidence varies by transplant modality; in autologous HCT, OUM afflicts 40–60% of recipients, whereas in allogeneic HCT, rates increase to 70–99%, which is attributable to intensified conditioning and graft-versus-host disease (GVHD) prophylaxis incorporating methotrexate [6]. 

Severe OUM significantly compromises patients’ quality of life through various mechanisms, including intractable oral pain, impaired nutritional intake, dysphagia, and dysarthria [7, 8]. These manifestations often necessitate escalation of analgesic interventions, including opioids, and dependence on total parenteral nutrition (TPN) to sustain caloric requirements [1, 7]. Additionally, OUM disrupts mucosal integrity, creating a portal for translocation of oral commensals and pathogens during neutropenia and thereby increasing risks of febrile neutropenia, bacteremia, and sepsis [9–12]. Seminal investigations have elucidated that severe mucositis confers a nearly fourfold elevation in infectious complications [12, 13]. This increased risk has economic ramifications, including incremental hospital charges surpassing $42,000 per episode, predominantly driven by protracted hospitalizations and intensified supportive care [1, 14, 15]. 

Hospital length of stay (LOS), delineated by discharge timing (discharge date minus admission date), represents a critical clinical and resource utilization metric in HCT [16, 17]. Autologous recipients are typically discharged within 10–20 days, whereas allogeneic cohorts often require 20–25 days or greater, owing to protracted engraftment, donor type, GVHD surveillance, and toxicity management [18, 19]. Delayed discharge frequently reflects underlying complications, such as severe OUM or infections, while concurrently heightening nosocomial exposure. This engenders a reciprocal dynamic: severe OUM may defer discharge via symptomatic burden and incipient infections, while prolonged hospitalization exacerbates toxicity through sustained immunosuppression and environmental pathogen exposure [11, 12]. 

Current literature mainly evaluates outcomes longitudinally or without stratification by discharge timing tailored to transplant type [10]. This makes it difficult to identify complication clustering in delayed-discharge cohorts. Stratifying by discharge timing—10–19 vs. 20–29 days in autologous HCT and 15–24 vs. 25–34 days in allogeneic HCT—creates an innovative lens to investigate whether protracted discharge signals increased systemic morbidity among those affected with OUM [10, 12]. 

Utilizing nationally representative discharge data, the present investigation characterizes OUM in HCT recipients stratified by discharge timing. We hypothesized that prolonged discharge cohorts (20–29 days for autologous and 25–34 days for allogeneic) would exhibit substantially elevated rates of both healthcare-associated complications such as septicemias and modality-specific morbidities, including coagulopathies, hypertension, and congestive heart failure, persisting after covariate adjustment. By prioritizing discharge-based stratification, this inquiry generates novel perspectives on the temporal aggregation of oral-systemic sequelae, informing precision supportive care to expedite safe discharge and reduce morbidity. This retrospective cohort study aimed to evaluate, among HCT recipients with documented OUM, whether prolonged hospital discharge timing—used as a pragmatic proxy for extended length of stay (LOS)—is associated with increased systemic complications. By focusing exclusively on patients who developed OUM and stratifying them by discharge timing, we sought to determine whether prolonged hospitalization identifies a high-risk subset of OUM patients with greater systemic morbidity.

To consider professionally-delivered oral care regimens as the gold standard for preventing systemic infections after HCT, a number of important questions must be addressed:

1. To what extent does prolonged hospital discharge timing—serving as a proxy for extended LOS—correlate with increased systemic complications, such as septicemia and healthcare-associated infections, among HCT recipients diagnosed with OUM?
2. Are there transplant-specific differences in the association between delayed discharge and adverse outcomes in patients with OUM?
3. Does the clustering of systemic morbidities (e.g., coagulopathies, hypertension, congestive heart failure) in prolonged-discharge cohorts persist after adjustment for demographic factors and comorbidity burden, suggesting a complication-driven prolongation of hospitalization?
4. Can discharge timing stratification (earlier vs. later) within a cohort of patients with documented OUM reveal patterns of morbidity that are obscured in unstratified comparisons?

## Methods

This retrospective cohort investigation utilized discharge abstracts from the 2021 National Inpatient Sample (NIS), the preeminent all-payer inpatient repository in the United States curated by the Healthcare Cost and Utilization Project (HCUP) under the Agency for Healthcare Research and Quality. The NIS implements a stratified probability sampling framework to yield nationally representative estimates, encompassing circa 20% of annual U.S. community hospital discharges. De-identification of records obviated institutional review board oversight.

### **Study Population**

Adult patients (≥ 18 years) hospitalized with leukemia or multiple myeloma undergoing autologous or allogeneic HCT complicated by ulcerative mucositis were identified via pertinent International Classification of Diseases, Tenth Revision (ICD-10) codes. Patients were then stratified according to discharge timing into modality-specific LOS categories: autologous HCT, discharged 10–19 vs. 20–29 days, and allogeneic HCT, discharged 15–24 vs. 25–34 days. Shorter discharges (0–10 days) served as referential comparators, devoid of adverse events. Baseline covariates abstracted from discharge records encompassed age, sex, race/ethnicity (White, Black, Hispanic, Asian/other), ZIP code-derived median household income quartile, primary payer (Medicare, Medicaid, private, self-pay/other), residence per the National Center for Health Statistics urban-rural schema, and comorbidity burden quantified by the weighted Elixhauser Comorbidity Index. Principal endpoints comprised systemic morbidities: healthcare-associated complications that comprise infectious complications (ICD codes provided) including septicemias (ICD codes provided), isolated septicemia (ICD codes provided), coagulopathies, hypertension, and congestive heart failure, identified through corresponding ICD-10 codes. Details of all ICD-10 codes used for cohort identification and outcome definition are provided in Supplementary Table S1.

### Inclusion and exclusion criteria

Inclusion criteria were (1) age ≥ 18 years, (2) diagnosis of leukemia or multiple myeloma, (3) autologous or allogeneic HCT procedure, and (4) documented diagnosis of oral ulcerative mucositis. Exclusion criteria included age < 18 years, absence of HCT procedure code, absence of oral ulcerative mucositis diagnosis, and discharges with missing key variables (rare in the NIS). Thus, no control group of patients without OUM was added.

### Comorbidity Assessment

Comorbidity burden was assessed using the weighted Elixhauser Comorbidity Index, a validated measure based on 30 comorbidity categories derived from administrative data [20, 21]. Higher scores indicate greater comorbidity burden.

Analyses accommodated NIS survey design, integrating stratification, clustering, and weighting for unbiased national inference. Descriptive metrics included weighted frequencies/percentages for categorical variables and means ± standard deviations for continuous variables. Inter-strata disparities were interrogated via survey-adjusted chi-square and weighted t-tests.

Multivariable survey-weighted logistic regression modeled adjusted odds ratios (aOR) with 95% confidence intervals for endpoints in prolonged vs. abbreviated discharge strata (the latter as the referent), adjusted for covariates age, sex, race/ethnicity, income quartile, payer, urban-rural designation, and Elixhauser score. Robust quasi-binomial variance estimation was applied. Computations employed R (version 3.6.1) with the survey package. The statistical significance threshold was bilateral, *p* < 0.05. Missing data, minimal in core NIS elements, underwent no imputation [20]. 

## Results

In autologous HCT recipients with OUM (weighted *n* = 1290), 78.3% (*n* = 1010) were discharged between 10 and 19 days and 21.7% (*n* = 280) between 20 and 29 days. The mean age was ~ 55 years in both groups (*p* = 0.61), but there were more females (51.8% vs. 40.6%; *p* = 0.17), and Asians/others (24.1% vs. 12.0%; *p* = 0.121) in the prolonged discharge cohort. Socioeconomic proxies (income, payer) and urban-rural distribution aligned (*p* > 0.4). The Elixhauser score was nonsignificantly elevated in prolonged discharge (17.0 vs. 15.0; *p* = 0.1) (Table 1).

**Table 1:** Baseline characteristics of autologous transplant ulcerative mucositis patients stratified by discharged patients for 10–19 days and 20–29 days

	Autologous transplant ulcerative mucositis patients discharged (10–19 days) (weighted)	Autologous transplant ulcerative mucositis patients discharged (20–29 days) (weighted).	*P*-value
*n*	1010 (78.29%)	280 (21.7%)	
AGE (mean (SD))	55.86 (13.74)	54.50 (15.62)	0.61
Sex (%)			0.17
Female	410 (40.6)	145 (51.8)	
RACE (%)			0.12
White	610 (61.0)	140 (51.9)	
Black	165 (16.5)	30 (11.1)	
Hispanic	105 (10.5)	35 (13.0)35 (13.0)	
Asian & Others	120 (12.0)	65 (24.1)	
Median household income (based on current year)			0.4
0-25th percentile	185.0 (18.6)	70.0 (25.0)	
26th to 50th percentile	250.0 (25.1)	50.0 (17.9)	
51st to 75th percentile	275.0 (27.6)	60.0 (21.4)	
76th to 100th percentile	285.0 (28.6)	100.0 (35.7)	
Expected primary payer (%)			0.79
Medicare	350.0 (34.8)	80.0 (28.6)	
Medicaid	100.0 (10.0)	35.0 (12.5)	
Private insurance	515.0 (51.2)	150.0 (53.6)	
Self-pay, No charge and other	40.0 (4.0)	15.0 (5.4)	
Patient Location: NCHS Urban-Rural Code (%)			0.52
“Central” counties of metro areas of > = 1 million population	350 (34.8)	80 (28.6)	
“Fringe” counties of metro areas of > = 1 million population	320 (31.7)	60 (21.4)	
Counties in metro areas of 250,000-999,999 population.	205 (20.3)	55 (19.6)	
Counties in metro areas of 50,000-249,999 population.	65 (6.4)	15 (5.4)	
Micropolitan counties & Not metropolitan or micropolitan counties	115 (11.4)	40 (14.3)	
Weighted Elixir score mean (SD)	14.98 (6.93)	17.02 (7.60)	0.1
Healthcare-associated complications (that include septicemias (%))	110 (10.9)	70 (25.0)	0.003
Septicemia (%)	60.0 (5.9)	55.0 (19.6)	0.001
Congestive Heart Failure (%)	20.0 (2.0)	40.0 (14.3)	< 0.001

Abbreviations: *SD* Standard deviation, *NCHS* National Center for Health Statistics, *$* United States’ Dollar, *UM* Ulcerative mucositis

Note: All frequencies and percentages are weighted

Morbidity escalated markedly in prolonged discharge: healthcare-associated complications including septicemias (25.0% vs. 10.9%; *p* = 0.003), isolated septicemia (19.6% vs. 5.9%; *p* = 0.001), and congestive heart failure (14.3% vs. 2.0%; *p* < 0.001). No events emerged in the 0–10 day discharge cohort (Table 2).

**Table 2:** Oral ulcerative mucositis outcomes among the length of stay groups, 10–19 LOS and 20–29 days (autologous transplant patients)

OUM-associated outcomes and other risks	Outcome estimates* (in the 20–29 days, compared to 10–19 days).	OUM-associated risk (unadjusted) of outcomes in the 10–19 and 20–29 days (number, percentage, and *p*-value)			OUM-associated risk (unadjusted) of outcomes during 0 days of admission and 10 days (number and percentages)	
		10–19 Days	20–29 Days	*P* value	0 LOS	10 LOS
Healthcare-associated complications that include septicemias	aOR: 3.09; 95%CI: 1.45–6.59	110 (10.9)	70 (25.0)	0.003	0 (0)	0 (0)
Septicemias alone	aOR: 4.68; 95% CI: 1.83–11.96	60.0 (5.9)	55.0 (19.6)	0.001	0 (0)	0 (0)
Other risks—developing congestive heart failure	aOR: 1.68; 95% CI: 2.44–11.58	20.0 (2.0)	40.0 (14.3)	< 0.001	0 (0)	0 (0)

Abbreviations: *Multivariate regression analysis (*aOR* adjusted odds ratio, *95% CI* 95% confidence interval) *OUM* Oral ulcerative mucositis

Adjusted regression confirmed the findings. Prolonged discharge conferred increased odds of healthcare-associated complications, including septicemias (aOR 3.09, 95%CI: 1.45–6.59), isolated septicemia (aOR 4.68, 95%CI 1.83–11.96), and congestive heart failure (aOR 1.68, 95% CI: 2.44–11.58) (Table 2).

Among allogeneic HCT recipients with OUM (weighted *n* = 885), 45.8% (*n* = 405) were discharged between 15 and 24 days and 54.2% (*n* = 480) between 25 and 34 days. Mean age was relatively similar between the cohorts (50 vs. 52 years; *p* = 0.43), although the prolonged discharge group had greater female representation (43.7% vs. 34.6%; *p* = 0.17) and a lower proportion of Whites (60.0% vs. 74.7%; *p* = 0.063). The Elixhauser score was significantly increased in prolonged discharge (7.7 vs. 5.7; *p* = 0.03) (Table 3).

**Table 3:** Baseline characteristics of allogeneic transplant ulcerative mucositis patients stratified by discharged patients for 15–24 days and 25–34 days

	Allogeneic transplant ulcerative mucositis patients discharged during 15–24 days (weighted)	Allogeneic transplant ulcerative mucositis patients discharged during 25–34 days (weighted)	*P*-value
*n*	405 (45.76%)	480 (54.24%)	
AGE (mean (SD))	49.84 (13.16)	51.59 (15.50)	0.43
Sex (%)			0.17
Female	140 (34.6)	210 (43.7)	
RACE (%)			0.06
White	295 (74.7)	285 (60.0)	
Black	5 (1.3)	40 (8.4)	
Hispanic	65 (16.5)	90 (18.9)	
Asian & Others	30 (7.6)	60 (12.6)	
Median household income (based on current year)			0.66
0-25th percentile	70.0 (17.3)	95.0 (19.8)	
26th to 50th percentile	85.0 (21.0)	95.0 (19.8)	
51st to 75th percentile	155.0 (38.3)	150.0 (31.2)	
76th to 100th percentile	95.0 (23.5)	140.0 (29.2)	
Expected primary payer (%)			0.08
Medicare	45.0 (11.3)	130.0 (27.1)	
Medicaid	50.0 (12.5)	75.0 (15.6)	
Private insurance	300.0 (75.0)	245.0 (51.0)	
Self-pay, No charge and other	5.0 (1.3)	30 (6.3)	
Patient Location: NCHS Urban-Rural Code (%)			0.296
“Central” counties of metro areas of > = 1 million population	100 (24.7)	175 (36.5)	
“Fringe” counties of metro areas of > = 1 million population	115 (28.4)	130 (27.1)	
Counties in metro areas of 250,000-999,999 population.	90 (22.2)	85 (17.7)	
Counties in metro areas of 50,000-249,999 population.	50 (12.3)	30 (6.3)	
Micropolitan counties & Not metropolitan or micropolitan counties	50 (12.3)	60 (12.5)	
Weighted Elixir score mean (SD)	5.7 (6.36)	7.69 (7.39)	0.03
Healthcare associated complications that includes septicemias (%)	30.0 (7.4)	85.0 (17.7)	0.04
Coagulopathies (%)	30.0 (7.4)	100.0 (20.8)	0.02
Hypertension (%)	120.0 (29.6)	220.0 (45.8)	0.01

Abbreviations: *SD* Standard deviation, *NCHS* National Center for Health Statistics, *$* United States’ Dollar, *UM* Ulcerative mucositis

Note: All frequencies and percentages are weighted

There were clear disparities between the allogenic HCT cohorts. Particularly, there were increased rates within the prolonged discharge group of healthcare-associated complications including septicemias (17.7% vs. 7.4%; *p* = 0.04), coagulopathies (20.8% vs. 7.4%; *p* = 0.02), and hypertension (45.8% vs. 29.6%; *p* = 0.01). There were no incidences of OUM in the 0–10 day allogenic discharge group (Table 4).

Multivariable modeling supported these findings. Prolonged discharge associated with OUM increased odds of healthcare-associated complications, including septicemias (aOR 3.3, 95%CI: 1.19–9.12), coagulopathies (aOR 3.47, 95%CI: 1.33–9.03), and hypertension (aOR 2.00, 95%CI: 1.05–3.82) (Table 4).

**Table 4:** Oral ulcerative mucositis-associated outcomes among the length of stay groups, 15–24 LOS and 25–35 days (allogeneic transplant patients)

OUM-associated outcomes and other risks	Outcome estimates* (in the 25–35 days, compared to 15–24 days).	OUM-associated risk (unadjusted) of outcomes in the 15–24 and 25–34 days (number, percentage, and *p*-value)			OUM-associated risk (unadjusted) of outcomes during 0 days of admission and 10 days (number and percentages)	
		15–24 Days	25–34 Days	*P* value	0 LOS	10 LOS
Healthcare-associated complications that include septicemias	aOR: 3.3; 95% CI: 1.19–9.12	30.0 (7.4)	85.0 (17.7)	0.04	0 (0)	0 (0)
coagulopathies	aOR: 3.47; 95% CI: 1.33–9.03	30.0 (7.4)	100.0 (20.8)	0.02	0 (0)	0 (0)
Hypertension	aOR: 2.00; 95% CI: 1.05–3.82	120.0 (29.6)	220.0 (45.8)	0.01	0 (0)	0 (0)

Abbreviations: *Multivariate regression analysis (*aOR* adjusted odds ratio, *95% CI* 95% confidence interval) *OUM* Oral ulcerative mucositis

Different systemic outcomes were examined in autologous and allogeneic HCT cohorts based on clinical plausibility and the patterns observed in the data. In autologous HCT, congestive heart failure emerged as a significant complication associated with prolonged discharge, whereas in allogeneic HCT, coagulopathies and hypertension were more prominent. This distinction reflects known differences in treatment exposures: autologous recipients often have greater prior exposure to cardiotoxic agents, while allogeneic recipients experience prolonged immunosuppression and higher rates of endothelial injury and drug-related toxicities.

Further, discharges within 0–10 days were analyzed as a separate reference category because this group had zero events for all examined systemic outcomes, consistent with rapid and uncomplicated recovery. Due to complete separation in the data, these cases were not included in the primary multivariable logistic regression models comparing 10–19/15–24 days versus prolonged discharge groups (Tables 2 and 4).

Collectively, these observations suggest an unfortunate reciprocal interplay between length of hospitalization and complications wherein HCT patients with OUM who have prolonged hospitalizations are at greater risk of complications, which can further increase their length of stay.

## Discussion

This nationally representative discharge-centric inquiry shows the significant morbidity associated with delayed discharge timing in HCT recipients afflicted by OUM [7, 8]. Across autologous and allogeneic modalities among patients with oral ulcerative mucositis (OUM), prolonged discharge timing (as a proxy for extended length of stay) is independently associated with higher rates of systemic complications, with adjusted odds ratios spanning 1.68–4.68 [12]. 

The autologous HCT group demonstrated escalation of septicemia-related complications and congestive heart failure within the prolonged discharge cohort (20–29 days) compared to the shorter one (10–19 days) [18, 22]. The near quintupling of isolated septicemia odds (aOR 4.68, 95%CI: 1.83–11.96) may highlight mucosal barrier compromise as a potential conduit for pathogen translocation amid neutropenia—a paradigm corroborated by meta-analytic synthesis demonstrating nearly fourfold infectious risk amplification [12, 23]. However, infections originating from origins other than the oral cavity or conditioning regimens may also increase hospital LOS and septicemias. The reason for increased odds of congestive heart failure remains unclear, as it is a relatively rare event, although it may be related to conditioning chemotherapy, impaired glucose tolerance, pre-existing cardiovascular risk factors, sepsis-mediated myocardial dysfunction, arrythmias such as atrial fibrillation, or TPN-associated fluid dysregulation in protracted oral intolerance [18, 24]. 

The transplant-specific differences in associated complications are noteworthy. Congestive heart failure in autologous HCT may be driven by anthracycline-related cardiotoxicity, fluid shifts from parenteral nutrition, or sepsis-induced cardiomyopathy in the setting of prolonged hospitalization. In allogeneic HCT, the higher incidence of coagulopathies and hypertension likely reflects endothelial damage from intensive conditioning, prolonged exposure to calcineurin inhibitors and corticosteroids, and the effects of graft-versus-host disease prophylaxis. These findings highlight that while septicemia-related complications are common to both groups, the additional morbidities associated with prolonged discharge differ by transplant type and underlying treatment exposures.

Interestingly, a greater proportion of allogeneic recipients required prolonged hospitalization, with over half not discharged until at least 25 days compared to 78% of autologous recipients being discharged within 10–19 days. This discrepancy is likely due to differences in time to engraftment and GVHD complications between the modalities. Prolonged discharge independently tripled septicemia-inclusive complications and coagulopathies while doubling hypertension [19, 22]. Increased coagulopathy is likely secondary to conditioning-induced endothelial perturbation, cytokine dysregulation, and infection-consumptive coagulopathy. The reason for increased hypertension is less clear but is perhaps driven by conditioning agents and immunosuppressants such as tacrolimus and cyclosporine, which promote fluid retention, vasoconstriction, and potential kidney damage [19, 24]. 

Central to this discussion is the interplay between OUM, length of hospitalization, and risk of complications. Severe OUM impedes timely discharge due to increased symptom burden and increased infection risk. Prolonged hospitalization, in turn, further intensifies nosocomial vulnerability and risk of complications [16, 17]. These relationships become more clear in a study such as this, which stratifies according to discharge time [16], and the inter-strata demographic similarity of this study serves to strengthen the relationship between discharge prolongation and related complications. Despite trends toward females, minority ethnicity, and comorbidity accrual in prolonged discharge, multivariable adjustment demonstrates robust associations [25, 26]. The lack of outcomes in short discharges within 0–10 days further substantiates the link between length of hospitalization and complication risk [10, 14]. 

Consistent with prior spending, standard pharmacoeconomic evaluations found nearly $42,000 in additional costs, mainly due to longer hospital stays [1, 14]. Interventional trials have shown that palifermin and photobiomodulation can improve severe OUM by 40–60%, which could possibly expedite discharge. Present data suggest that such interventions, along with intensified oral hygiene and preemptive microbials, should be prioritized for those with the potential for prolonged hospitalizations [11, 27]. Multidisciplinary algorithms integrating hematology, oral medicine, infectious diseases, and nutrition may improve LOS and therefore morbidity. Measures to prevent or lessen the severity of OUM would also financially benefit the healthcare system, and quality metric bundling of supportive modalities may catalyze their dissemination.

The post-transplant phase following HCT represents a prolonged period of immunological vulnerability, marked by gradual hematopoietic reconstitution, lingering mucosal fragility, and persistent susceptibility to opportunistic pathogens. The present investigation, which reveals substantially elevated systemic complications among patients with OUM experiencing delayed discharge, highlights the critical need for comprehensive, multidisciplinary oral care that extends well beyond neutrophil engraftment. Although pre-transplant dental evaluation and sanitation may minimize preexisting infectious foci, the post-transplant period requires systematic oral management to preserve mucosal barrier integrity, ameliorate mucositis sequelae, and hopefully reduce morbidity [22, 23]. 

Current clinical practice guidelines from the Multinational Association of Supportive Care in Cancer and the International Society of Oral Oncology (MASCC/ISOO) strongly advocate for structured basic oral care protocols as the cornerstone of mucositis prevention and mitigation in those undergoing HCT [28]. These protocols involve daily gentle mechanical cleansing with ultra-soft toothbrushes, use of alcohol-free fluoride-containing rinses, and application of moisturizing agents to counteract xerostomia—a common post-conditioning sequela that exacerbates ulceration and facilitates microbial overgrowth [29, 30]. Meta-analyses of randomized trials confirm that multifaceted basic oral care regimens, incorporating saline or sodium bicarbonate rinses, significantly reduce mucositis severity, pain scores, and secondary infection rates in HCT recipients, supporting the need for integrated multi-disciplinary care teams [29, 30]. 

Emerging therapeutic modalities offer additional promise for post-transplant oral stewardship [11, 27]. Photobiomodulation therapy (PBMT), also known as low-level laser therapy, has received strong guideline endorsement for prophylactic use in high-risk conditioning regimens. By promoting epithelial proliferation, modulating inflammatory cytokines, and enhancing tissue repair, PBMT reduces the incidence and duration of severe mucositis, with sustained benefits observable into the early post-engraftment phase. Similarly, oral cryotherapy—administration of ice chips during cytotoxic infusions—reduces mucositis in melphalan-containing protocols, producing protective effects that persist post-transplant [1, 11]. Professional dental engagement remains indispensable before, during, and after engraftment. While profound neutropenia precludes invasive interventions, periodic oral examinations by trained dental hygienists or oral medicine specialists enable timely identification of secondary infections, oral candidiasis, or herpes virus reactivation, which are frequent complications in the immunocompromised host [12]. Population-based studies suggest that structured peri-transplant oral care correlates with reduced post-HCT infectious events, although direct effects on mucositis duration remain variable. In allogeneic recipients, where chronic graft-versus-host disease (cGVHD) commonly manifests with oral lichenoid changes, sclerotic features, or salivary gland dysfunction months to years post-transplant, ongoing oral care and dental surveillance is essential to prevent secondary caries, periodontal progression, mucosal fibrosis, and rare but serious secondary malignancies [9, 24]. 

Patient-centered education forms an integral component of effective post-transplant oral care. Empowering recipients with individualized hygiene guidance tailored to thrombocytopenia, GVHD-related fragility, and xerostomia fosters adherence and self-management [29, 30]. Dietary counseling emphasizing soft, bland, non-acidic foods minimizes mechanical irritation, and multimodal analgesia, including topical agents such as doxepine or lidocaine formulations (benzydamine in countries where available) alleviates discomfort, promotes oral intake, and potentially reduces reliance on total parenteral nutrition [31]. Despite compelling evidence, barriers to optimal post-transplant oral care persist, including inconsistent protocol implementation across centers, limited access to specialized oral medicine services, and variable reimbursement structures. Implementation science studies, particularly in pediatric HCT populations, demonstrate that bundled evidence-based oral care interventions enhance compliance and clinical outcomes. Seamless integration of oral care into multidisciplinary transplant care pathways—involving hematologists, nursing staff, infectious disease specialists, and dental professionals—holds potential to interrupt the vicious cycle of mucositis, infection, and hospitalization prolongation observed in this study [28, 29]. 

Given our findings of clustered morbidity in prolonged-discharge cohorts, intensified post-transplant oral interventions warrant prioritization in patients exhibiting delayed recovery [32]. Such strategies may not only mitigate systemic complications but also facilitate earlier safe discharge and have implications throughout survivorship. Prospective clinical trials evaluating comprehensive oral care bundles—incorporating PBMT, professional monitoring, and enhanced patient education—with endpoints encompassing infection incidence, quality-of-life measures, and healthcare utilization are needed. These efforts will translate observational insights into actionable improvements, ultimately refining supportive care paradigms and reducing the substantial burden of post-HCT oral morbidity [12, 27]. 

The study’s deliberate restriction to patients with documented OUM—and its comparison of outcomes between earlier versus later discharge timing within this affected group—represents a methodologically robust and clinically meaningful design choice, rather than a limitation. This approach strengthens the inference that prolonged hospitalization (delayed discharge) reflects complication severity and clustering in mucositis-afflicted patients, rather than merely comparing mucositis-positive versus mucositis-negative cases [10–12].

- Focused examination of mucositis-related morbidity burden — By including only patients with OUM, the study isolates the subpopulation most vulnerable to systemic complications. Non-ulcerative mucositis (e.g., grades 1–2, manifesting as erythema or mild soreness without frank ulceration) typically entails less severe pain, preserved oral intake, and minimal infection risk through intact mucosal barriers. In contrast, ulcerative mucositis (grades 3–4) breaches epithelial integrity, serving as a direct portal for bacterial translocation during neutropenia—a key driver of septicemia, healthcare-associated infections, and secondary organ dysfunction [33, 34]. Including non-ulcerative cases would dilute effect sizes and obscure the specific, high-impact sequelae of barrier-disrupting ulceration, which the study explicitly targets [22, 23].
- Discharge timing as a proxy for complication-driven hospital prolongation — The core hypothesis posits a bidirectional relationship: severe OUM contributes to delayed discharge (via pain, nutritional failure, and infections), while prolonged hospitalization exacerbates nosocomial risks and cumulative toxicity [16, 17]. Stratifying within OUM-positive patients by discharge timing (earlier vs. later) directly tests whether more severe or complicated mucositis courses manifest as extended stays. This yields greater clinical relevance than a simple mucositis-present-versus-absent comparison, which would conflate mild (non-ulcerative) cases with severe ones and fail to capture temporal clustering of morbidity. The observed 3–4.7-fold adjusted risks for septicemia and other outcomes in prolonged-discharge OUM patients strongly support that delayed discharge serves as a real-world marker of mucositis severity and complication accrual [22, 35].
- Avoidance of confounding by mild or asymptomatic cases — Non-ulcerative mucositis often resolves rapidly with basic supportive care and rarely precipitates systemic events or hospitalization extensions. Including such cases would introduce heterogeneity, potentially attenuating associations and underestimating the true burden in the high-risk ulcerative subgroup [36]. Administrative data (e.g., NIS ICD-10 coding) further justify this exclusion: codes for mucositis typically capture clinically significant (often ulcerative) disease warranting documentation, whereas mild erythema may be undercoded or omitted. Thus, the cohort inherently enriches for impactful cases [10–12].
- Alignment with clinical and research priorities — Supportive care guidelines (MASCC/ISOO) and interventional trials (e.g., palifermin, photobiomodulation) prioritize prevention/mitigation of severe/ulcerative mucositis due to its dominant contribution to morbidity, costs, and quality-of-life impairment. By focusing on this subgroup and using discharge timing to stratify severity indirectly, the study generates actionable insights for resource-intensive patients most likely to benefit from intensified oral care, infection prophylaxis, or early intervention—precisely those with prolonged stays [12, 14].

Thus, our examination sharpens the focus on the clinically dominant severe phenotype, leverages discharge timing as a pragmatic severity proxy, minimizes confounding by mild disease, and enhances translational relevance for high-burden scenarios in HCT supportive care [37]. 

### Study Design Considerations

The present study was deliberately restricted to patients with documented oral ulcerative mucositis rather than comparing OUM-positive versus OUM-negative patients. This approach was chosen because ulcerative mucositis constitutes the biologically and clinically most relevant phenotype, directly disrupting the mucosal barrier and facilitating bacterial translocation during neutropenia. Milder (non-ulcerative) or absent mucositis cases generally carry a substantially lower risk of systemic complications and rarely result in prolonged hospitalization. By focusing exclusively on patients with OUM and stratifying them according to discharge timing, the analysis tests whether prolonged hospitalization serves as a real-world marker of greater complication severity and clustering within this high-risk subgroup. Although evaluating OUM as an independent predictor of prolonged LOS (followed by LOS as a predictor of complications) would be a valuable complementary analysis, such a stepwise approach was outside the scope of this manuscript. Future studies may usefully address this broader question.

Limitations warrant acknowledgment. NIS retrospective architecture hinges on administrative coding, potentially under-identifying subclinical mucositis or omitting severity gradation (e.g., WHO criteria). Inferential causality remains constrained; unmeasured confounding (e.g., regimen specifics, prophylaxis) persists despite adjustment. Inpatient confinement precludes post-discharge sequelae. Select estimates evince interval breadth, reflective of strata dimensionality notwithstanding weighting.

Study merits include expansive national representativeness facilitating modality-specific discharge stratification—infrequent in unicentric cohorts—and stringent survey analytics mitigating bias. Discharge timing as a pragmatic endpoint augments translational fidelity. Prospective avenues encompass clinical gradation validation, regimen granularity incorporation, and randomized protracted-discharge interventional trials. Early OUM biomarker/machine learning prognostication could enable bespoke risk stratification and prophylaxis. Further future examination through all grade non-mucositis cases might provide execellent comparisons to mitigate oral-systemic associations.

In conclusion, delayed discharge timing in ulcerative mucositis-complicated HCT robustly prognosticates—and likely propagates—severe oral-systemic complications. These findings impel discharge-stratified supportive paradigms to improve modifiable hazards, hasten safe discharge, and optimize clinical/fiscal outcomes in this susceptible population.

## Supplementary Information

Below is the link to the electronic supplementary material.

Supplementary Material 1 (DOCX 18.8 KB)

## Data Availability

We used data from the United States’ 2021 National Inpatient Sample database obtained from Healthcare Cost and Utilization Project (HCUP) of the Agency for Healthcare Research and Quality (AHRQ).
