# Supplementary material for: Stratified discharge timing within mucositis cases and its association with systemic complications in hematopoietic stem cell transplant recipients: insights from a national inpatient study
Source: Clin Oral Investig. 2026 Jul 21;30(8):353. doi: 10.1007/s00784-026-07037-w (PMC13388775; doi:10.1007/s00784-026-07037-w)
Supplement: Supplementary file 1 — Supplementary Material 1 (DOCX 18.8 KB) [file 784_2026_7037_MOESM1_ESM.docx]

**Allogeneic bone marrow transplant ICD PCS codes**

| **30230G2** | Transfusion of Allogeneic Related Bone Marrow into Peripheral Vein, Open Approach |
| --- | --- |
| **30230G3** | Transfusion of Allogeneic Unrelated Bone Marrow into Peripheral Vein, Open Approach |
| **30230G4** | Transfusion of Allogeneic Unspecified Bone Marrow into Peripheral Vein, Open Approach |
| **30230X0** | Transfusion of Autologous Cord Blood Stem Cells into Peripheral Vein, Open Approach |
| **30230X2** | Transfusion of Allogeneic Related Cord Blood Stem Cells into Peripheral Vein, Open Approach |
| **30230X3** | Transfusion of Allogeneic Unrelated Cord Blood Stem Cells into Peripheral Vein, Open Approach |
| **30230X4** | Transfusion of Allogeneic Unspecified Cord Blood Stem Cells into Peripheral Vein, Open Approach |
| **30230Y2** | Transfusion of Allogeneic Related Hematopoietic Stem Cells into Peripheral Vein, Open Approach |
| **30230Y3** | Transfusion of Allogeneic Unrelated Hematopoietic Stem Cells into Peripheral Vein, Open Approach |
| **30230Y4** | Transfusion of Allogeneic Unspecified Hematopoietic Stem Cells into Peripheral Vein, Open Approach |
| **30233G2** | Transfusion of Allogeneic Related Bone Marrow into Peripheral Vein, Percutaneous Approach |
| **30233G3** | Transfusion of Allogeneic Unrelated Bone Marrow into Peripheral Vein, Percutaneous Approach |
| **30233G4** | Transfusion of Allogeneic Unspecified Bone Marrow into Peripheral Vein, Percutaneous Approach |
| **30233X2** | Transfusion of Allogeneic Related Cord Blood Stem Cells into Peripheral Vein, Percutaneous Approach |
| **30233X3** | Transfusion of Allogeneic Unrelated Cord Blood Stem Cells into Peripheral Vein, Percutaneous Approach |
| **30233X4** | Transfusion of Allogeneic Unspecified Cord Blood Stem Cells into Peripheral Vein, Percutaneous Approach |
| **30233Y2** | Transfusion of Allogeneic Related Hematopoietic Stem Cells into Peripheral Vein, Percutaneous Approach |
| **30233Y3** | Transfusion of Allogeneic Unrelated Hematopoietic Stem Cells into Peripheral Vein, Percutaneous Approach |
| **30233Y4** | Transfusion of Allogeneic Unspecified Hematopoietic Stem Cells into Peripheral Vein, Percutaneous Approach |
| **30240G2** | Transfusion of Allogeneic Related Bone Marrow into Central Vein, Open Approach |
| **30240G3** | Transfusion of Allogeneic Unrelated Bone Marrow into Central Vein, Open Approach |
| **30240G4** | Transfusion of Allogeneic Unspecified Bone Marrow into Central Vein, Open Approach |
| **30240X0** | Transfusion of Autologous Cord Blood Stem Cells into Central Vein, Open Approach |
| **30240X2** | Transfusion of Allogeneic Related Cord Blood Stem Cells into Central Vein, Open Approach |
| **30240X3** | Transfusion of Allogeneic Unrelated Cord Blood Stem Cells into Central Vein, Open Approach |
| **30240X4** | Transfusion of Allogeneic Unspecified Cord Blood Stem Cells into Central Vein, Open Approach |
| **30240Y2** | Transfusion of Allogeneic Related Hematopoietic Stem Cells into Central Vein, Open Approach |
| **30240Y3** | Transfusion of Allogeneic Unrelated Hematopoietic Stem Cells into Central Vein, Open Approach |
| **30240Y4** | Transfusion of Allogeneic Unspecified Hematopoietic Stem Cells into Central Vein, Open Approach |
| **30243G2** | Transfusion of Allogeneic Related Bone Marrow into Central Vein, Percutaneous Approach |
| **30243G3** | Transfusion of Allogeneic Unrelated Bone Marrow into Central Vein, Percutaneous Approach |
| **30243G4** | Transfusion of Allogeneic Unspecified Bone Marrow into Central Vein, Percutaneous Approach |
| **30243X2** | Transfusion of Allogeneic Related Cord Blood Stem Cells into Central Vein, Percutaneous Approach |
| **30243X3** | Transfusion of Allogeneic Unrelated Cord Blood Stem Cells into Central Vein, Percutaneous Approach |
| **30243X4** | Transfusion of Allogeneic Unspecified Cord Blood Stem Cells into Central Vein, Percutaneous Approach |
| **30243Y2** | Transfusion of Allogeneic Related Hematopoietic Stem Cells into Central Vein, Percutaneous Approach |
| **30243Y3** | Transfusion of Allogeneic Unrelated Hematopoietic Stem Cells into Central Vein, Percutaneous Approach |
| **30243Y4** | Transfusion of Allogeneic Unspecified Hematopoietic Stem Cells into Central Vein, Percutaneous Approach |
| **30250G1** | Transfusion of Nonautologous Bone Marrow into Peripheral Artery, Open Approach |
| **30250X0** | Transfusion of Autologous Cord Blood Stem Cells into Peripheral Artery, Open Approach |
| **30250X1** | Transfusion of Nonautologous Cord Blood Stem Cells into Peripheral Artery, Open Approach |
| **30250Y1** | Transfusion of Nonautologous Hematopoietic Stem Cells into Peripheral Artery, Open Approach |
| **30260G1** | Transfusion of Nonautologous Bone Marrow into Central Artery, Open Approach |
| **30260X0** | Transfusion of Autologous Cord Blood Stem Cells into Central Artery, Open Approach |
| **30260X1** | Transfusion of Nonautologous Cord Blood Stem Cells into Central Artery, Open Approach |
| **30260Y1** | Transfusion of Nonautologous Hematopoietic Stem Cells into Central Artery, Open Approach |
| **30253X1** | Transfusion of Nonautologous Cord Blood Stem Cells into Peripheral Artery, Percutaneous Approach |
| **30253G1** | Transfusion of Nonautologous Bone Marrow into Peripheral Artery, Percutaneous Approach |

**30263G1** Transfusion of Nonautologous Bone Marrow into Central Artery, Percutaneous Approach

**30263X1** Transfusion of Nonautologous Cord Blood Stem Cells into Central Artery, Percutaneous Approach

**30263Y1** Transfusion of Nonautologous Hematopoietic Stem Cells into Central Artery, Percutaneous Approach.

**Autologous bone marrow transplant ICD PCS codes**

| **30230AZ** | Transfusion of Embryonic Stem Cells into Peripheral Vein, Open Approach |
| --- | --- |
| **30230G0** | Transfusion of Autologous Bone Marrow into Peripheral Vein, Open Approach |
| **30230X0** | Transfusion of Autologous Cord Blood Stem Cells into Peripheral Vein, Open Approach |
| **30230Y0** | Transfusion of Autologous Hematopoietic Stem Cells into Peripheral Vein, Open Approach |
| **30240AZ** | Transfusion of Embryonic Stem Cells into Central Vein, Open Approach |
| **30240G0** | Transfusion of Autologous Bone Marrow into Central Vein, Open Approach |
| **30240X0** | Transfusion of Autologous Cord Blood Stem Cells into Central Vein, Open Approach |
| **30240Y0** | Transfusion of Autologous Hematopoietic Stem Cells into Central Vein, Open Approach |

| **30233AZ*** | Transfusion of Embryonic Stem Cells into Peripheral Vein, Percutaneous Approach |
| --- | --- |
| **30233G0*** | Transfusion of Autologous Bone Marrow into Peripheral Vein, Percutaneous Approach |
| **30233X0*** | Transfusion of Autologous Cord Blood Stem Cells into Peripheral Vein, Percutaneous Approach |
| **30233Y0*** | Transfusion of Autologous Hematopoietic Stem Cells into Peripheral Vein, Percutaneous Approach |
| **30243AZ*** | Transfusion of Embryonic Stem Cells into Central Vein, Percutaneous Approach |
| **30243G0*** | Transfusion of Autologous Bone Marrow into Central Vein, Percutaneous Approach |
| **30243X0*** | Transfusion of Autologous Cord Blood Stem Cells into Central Vein, Percutaneous Approach |
| **30243Y0*** | Transfusion of Autologous Hematopoietic Stem Cells into Central Vein, Percutaneous Approach |

**Septicemia ICD CM codes**

A021, A227, A267, A327, A400-401, A403, A408-409, A4101-4102, A411-414, A4150-4153, A4159, A4181, A4189, A419, A427, A5486, B377, R6520, R6521.

**Oral Ulcerative Mucositis ICD CM Code (K1231)**

**ICD-10-CM billable codes for Healthcare-associated infection (HAIs)**

**ventilator-associated pneumonia (VAP)** - J95851

**central line-associated bloodstream infection (CLABSI)** - T80211A", "T80211D", "T80211S", "T80212A", "T80212D", "T80212S", "T80218A", "T80218D", "T80218S", "T80219A", "T80219D", "T80219S", "T8022XA", "T8022XD", "T8022XS", "T8029XA", "T8029XD", "T8029XS".

**catheter-associated urinary tract infection (CAUTI)** - "T83510A", "T83510D", "T83510S", "T83511A", "T83511D", "T83511S", "T83512A","T83512D", "T83512S", "T83518A", "T83518D", "T83518S", "T83590A", "T83590D", "T83590S", "T83591A", "T83591D", "T83591S", "T83592A", "T83592D", "T83592S", "T83593A", "T83593D", "T83593S", "T83598A", "T83598D", "T83598S".

and **Clostridium difficile infection (CDI)** - "A0471", "A0472"

**ICD-10-CM billable codes for Healthcare-associated complications (HAIs and Septicemias)**

**Septicemia ICD codes**

A021, A227, A267, A327, A400-401, A403, A408-409, A4101-4102, A411-414, A4150-4153, A4159, A4181, A4189, A419, A427, A5486, B377, R6520, R6521.

**ventilator-associated pneumonia (VAP)** - J95851

**central line-associated bloodstream infection (CLABSI)** - T80211A", "T80211D", "T80211S", "T80212A", "T80212D", "T80212S", "T80218A", "T80218D", "T80218S", "T80219A", "T80219D", "T80219S", "T8022XA", "T8022XD", "T8022XS", "T8029XA", "T8029XD", "T8029XS".

**catheter-associated urinary tract infection (CAUTI)** - "T83510A", "T83510D", "T83510S", "T83511A", "T83511D", "T83511S", "T83512A","T83512D", "T83512S", "T83518A", "T83518D", "T83518S", "T83590A", "T83590D", "T83590S", "T83591A", "T83591D", "T83591S", "T83592A", "T83592D", "T83592S", "T83593A", "T83593D", "T83593S", "T83598A", "T83598D", "T83598S".

and **Clostridium difficile infection (CDI)** - "A0471", "A0472"
